# Genetic Diversity Analysis of *Hypsizygus marmoreus* with Target Region Amplification Polymorphism

**DOI:** 10.1155/2014/619746

**Published:** 2014-06-09

**Authors:** Chengshu Qiu, Wenjuan Yan, Wangqiu Deng, Bin Song, Taihui Li

**Affiliations:** ^1^Guangdong Provincial Key Laboratory of Microbial Culture Collection and Application, Guangdong Open Laboratory of Applied Microbiology, State Key Laboratory of Applied Microbiology South China, Guangdong Institute of Microbiology, Guangzhou 510070, China; ^2^Department of Biology, Chengdu Normal University, Chengdu 611130, China

## Abstract

*Hypsizygus marmoreus* is an industrialized edible mushroom. In the present paper, the genetic diversity among 20 strains collected from different places of China was evaluated by target region amplification polymorphism (TRAP) analysis; the common fragment of TRAPs was sequenced and analyzed. Six fixed primers were designed based on the analysis of *H. marmoreus* sequences from GenBank database. The genomic DNA extracted from *H. marmoreus* was amplified with 28 TRAP primer combinations, which generated 287 bands. The average of amplified bands per primer was 10.27 (mean polymorphism is 69.73%). The polymorphism information content (PIC) value for TRAPs ranged from 0.32 to 0.50 (mean PIC value per TRAP primer combination is 0.48), which indicated a medium level of polymorphism among the strains. A total of 36 sequences were obtained from TRAP amplification. Half of these sequences could encode the known or unknown proteins. According to the phylogenetic analysis based on TRAP result, the 20 strains of *H. marmoreus* were classified into two main groups.

## 1. Introduction

*Hypsizygus marmoreus* (Peck) H. E. Bigelow is an industrialized edible mushroom [1] and has become one of the most popular edible and medicinal mushrooms in East Asia because of its rich nutrition and medicinal value [2–7]. Quite a lot of studies on this edible fungus were focused on the nutrient substances, physiological characters, and healthcare functions for human, but genetic researches were not sufficient. The characteristics of strains are very important for the production output and quality of the cultivated mushrooms. Molecular markers can directly reflect the genetic polymorphisms in DNA levels and have been widely used to analyze the genetic diversities, construct the genetic linkage maps, identify strains, and assist in breeding [8, 9]. In the previous studies, the amplified fragment length polymorphism (AFLP) markers and sequence characterized amplified region (SCAR) markers were developed to discriminate the strains and analyse the genetic divergences among the strains of* H. marmoreus* [10, 11]. Although a few studies on molecular markers of* H. marmoreus* have been carried out, the tested strains and the applied analysis methods were limited [12–14].

Target region amplification polymorphism (TRAP) was developed on the basis of sequence-relation amplification polymorphism (SRAP) [15]. Compared with other marker techniques, TRAP has the advantages of easy operation, high stability and repeatability, strong targeting ability, and rich polymorphism information [16, 17]. It had been used to analyze the genetic diversity of* Lentinula edodes* [18], construct the genetic linkage map of* Lentinula edodes* [9], and analyze the relationship between phenotypic and genotypic traits of* Auricularia auricula-judae* [8]. However, it has not been applied in* H. marmoreus*.

In this study, the strains of* H. marmoreus* were extensively collected in China; the genetic diversities were evaluated with TRAP technique, and the genes of serine protease and fungal intracellular laccase were sequenced [19, 20]. The result will provide important genetic information for further studies on strain conservation, identification or discrimination of the strains, and new functional gene discovery.

## 2. Material and Methods

### 2.1. Fungus Materials

Twenty strains of* H. marmoreus* were collected from the Agricultural Culture Collection of China (Beijing, 6), provincial academies of agricultural science (Fujian, 2; Hunan, 2; Sichuan, 1), institutes of edible fungi and mushrooms (Gaoyou, 1; Mianyang, 1; Sanming, 1), Changbaishan (Jinlin, 1), and mushroom enterprises in different places (Shanghai, 2; Shandong, 1; Guangdong, 1; Hubei, 1) (Supplementary File 1 (Table S1) available online at http://dx.doi.org/10.1155/2014/619746), including 7 storage strains and 13 commercial strains (Supplementary File 1 (Table S1)). The mycelia of the strains were cultured on potato sucrose agar (PSA) medium at 25°C.

### 2.2. DNA Extraction

Genomic DNA was extracted from fresh mycelium using the Sangon Fungus Genomic DNA Extraction Kit (Sangon Biotech Co., Ltd., Shanghai). DNA concentration and purity were determined by spectrophotometry (BioSpec-nano, Shimadzu, Japan) and electrophoresis in 1.4% agarose gels with known DNA marker.

### 2.3. Primers Design and TRAP Analysis

Initially 4 sequences of the* H. marmoreus* were selected to design the fixed primers [19, 21, 22]. All fixed primers were designed by web-based software “Primers 3” [23, 24] (http://frodo.wi.mit.edu/). The arbitrary primers were designed according to [16, 25, 26]. All fixed primers and arbitrary primers were synthesized by Sangon (Sangon Biotech Co., Ltd., Shanghai, China). The information of all primers was showed in Supplementary File 1 (Table S2).

PCR was performed in a 25 *μ*L reaction mixture containing 30 ng template DNA, 1.5 *μ*L 2.5 mM dNTP, 1.5 *μ*L 25 mM MgCl_2_, 1.5 *μ*L 10× buffer (0.1 mM EDTA, 10.0 mM KCl, and 20 mM Tris-HCl in pH 8.0), 0.75 *μ*L 10 mM primers, and 0.5 *μ*L 2.5 U of Taq DNA polymerase (Tiangen Biotech, Beijing, China). A blank control and three negative controls were designed to improve the accuracy and reliability of TRAP results. The three negative controls contained DNA from* Coprinus comatus*, no primers, and blank control. To ensure the specificity of the experiment, only the bands specific to* H. marmoreus* were counted; that is, the bands amplified from both strains of* H. marmoreus *and not in strain of* C. comatus* were excluded from analyses. Controls without primers were used to detect and eliminate reagent contamination.

Amplification was performed in a Biometra TProfessional Standard (Biometra, GmbH, Germany). The TRAP-PCR reaction was modified based on Hu and Vick [15] as follows: 94°C for 10 min, 10 cycles of 94°C for 1 min, 35°C for 1 min, and 72°C for 2 min 30 s, then 30 cycles of 94°C for 1 min, 51.5°C for 1 min, and 72°C for 2 min 30 s, and a final extension step of 10 min at 72°C, and then stored at 4°C.

Amplified products were electrophoresed in 2% agarose gel with 0.5 Tris/Borate/EDTA buffer at 120 V for 45 min and stained with ethidium bromide (0.5 g/mL). Gels with amplification fragments were visualized and photographed under ultraviolet light using a GE Image Quant Digital Imaging System (GE Healthcare Bio-Sciences AB, Sweden). The DL2000 DNA ladder was used as a marker (Tiangen Biotech, Beijing, China).

### 2.4. Data Analysis

TRAP amplification products were compared with DNA ladder markers and scored using a binary code (present, 1; absent, 0) of each genotype using manual score; then the data of matrix were recorded in excel files for further analysis. Only well-separated bands with a high intensity of polymorphism were selected as markers. Pairwise comparisons were calculated using Jaccard's coefficient [27]. The similarity values were used to generate a consensus tree using the unweighted pair group method with arithmetic mean (UPGMA) algorithm [28]. Analyses were performed with NTSYS-pc version 2.1 [29]. The PIC, MI, qualitative nature of data, and effective MI were calculated as follows.

PIC values measure the information of a given DNA marker, PIC_*i*_ = 1 − ∑(*P*_*ij*_)^2^, where *P*_*ij*_ is the frequency of the *i*th pattern revealed by the *j*th primer summed across all patterns revealed by the primers [30, 31]. The MI is the product of the total number of loci per primer (*n*). The MI is calculated for each ISSR primer as MI = PIC × *ηβ*, where PIC is the mean PIC value, *η* is the number of bands, and *β* is the proportion of polymorphism [32].

### 2.5. Sequencing Analyses

The common fragments only in all TRAP-PCR amplifications with* H. marmoreus* were extracted from agarose gels using spin column DNA extraction kit and cloned into pMD19-T vector (TaKaRa, Dalian, China). The clones were sequenced by Beijing Genomics Institute (BGI) after PCR check using universal primers (GV-M, 5′-GAGCGGATAACAATTTCACACAGG-3′ and M13–47, 5′-CGCCAGGGTTTTCCCAGTCACGAC-3′). Sequences were assembled using program of SeqMan (software of DNASTAR package) and submitted to GenBank.

The homology of sequences was predicted by web-based tool BLAST search [33–36]. The extrons or introns of sequence analyses were predicted by web-based program FENGE SH [37–39] (http://linux1.softberry.com/berry.phtml).

## 3. Results and Discussion

### 3.1. TRAP Analysis

**Figure 1:**
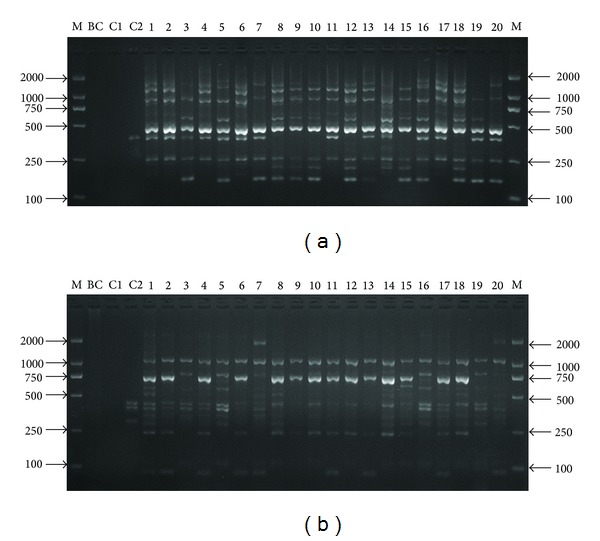
Fragment profiles of* Hypsizygus marmoreus* generated by primer combination of TRAP. ((a) EU94L1-EM9, (b) GQ76R1-ME1, BC, C1, and C2 correspond to blank control, no primers, and* Coprinus comatus*; the spots from 1 to 20 correspond to the strains of* H. marmoreus* (see Supplementary Table S1)).

After primary screening, 6 fixed primers and 20 arbitrary primers were selected to second screening. Twenty-eight primer combinations could produce clear bands with good polymorphisms and reproducibility after second screening (Table 1). These primer combinations generated a total of 287 scorable fragments, 202 of which were polymorphic. The average of fragments per primer combination was 10.27, and the total of fragments amplified by each TRAP primer combination ranged from 4 to 17. The percentages of polymorphic fragments ranged from 33.3% (JN00L1-ME11) to 100% (JN00R1-ME6) with a mean of 69.73% (Table 1). The size of the detected fragments ranged from 100 to 2100 bp (Table 1, Figure 1).

The PIC values for TRAP markers ranged from 0.32 to 0.50, and the mean PIC value per TRAPs was 0.48. The MI for TRAPs ranged from 1.92 to 8.50, and the mean MI per ISSR was 4.94 (Table 1).

The PIC value provides an evaluation of the discriminating power of the TRAP marker to polymorphism. Generally, PIC value between 0.00 and 0.25 implied a very low genetic diversity among samples, PIC value between 0.25 and 0.50 shows a medium level of genetic diversity, and PIC value above 0.50 suggests a high level of genetic diversity [30, 31, 40, 41]. In this study, the PIC values for TRAP markers ranged from 0.32 to 0.5, with a mean value of 0.48 (Table 1), which indicated a medium level of genetic diversity among the* H. marmoreus* strains.

### 3.2. Phylogenetic Analysis and Principal Coordinates Analysis (PCoA) Based on TRAP Markers

**Figure 2:**
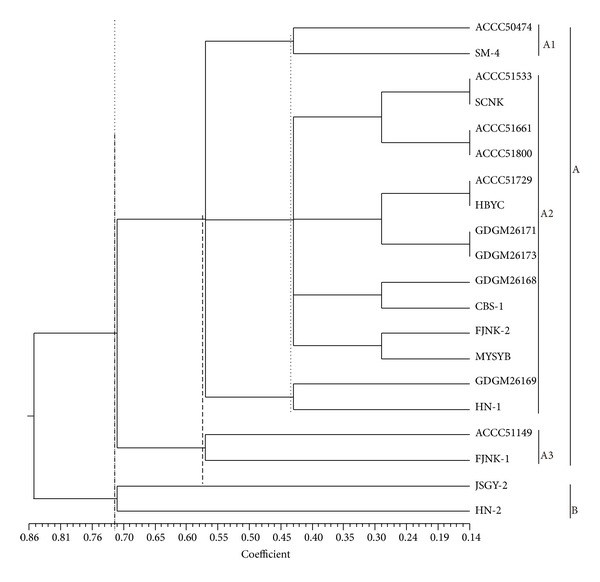
Phylogenetic tree based on TRAP data. The* Hypsizygus marmoreus* strains were classified into two subgroups at Jaccard's similarity coefficient of 0.718. Group B included strains of JSGY-2 and HN-2. Group A had the remaining 18 strains. Group A was classified into 3 subgroups at Jaccard's coefficients of 0.575 and 0.435.

A phylogenetic tree was constructed by UPGMA cluster analysis using 287 TRAP fragments from the 20 strains of* H. marmoreus* (Figure 2). All strains were classified into two clusters (at Jaccard's similarity coefficient of 0.718): cluster A consisted of 18 strains of* H. marmoreus* and JSGY-2 and HN-2 with white basidiocarps were members of cluster B. Cluster A formed three subgroups, A1, A2, and A3, at Jaccard's similarity coefficients of 0.575 and 0.435. Both subgroups A1 and A3 included two strains, ACCC5047 and SM-4, in subgroup A1 and ACCC51149 and FJNK-1 in subgroup A3; the remaining strains were clustered in subgroup A2.

**Figure 3:**
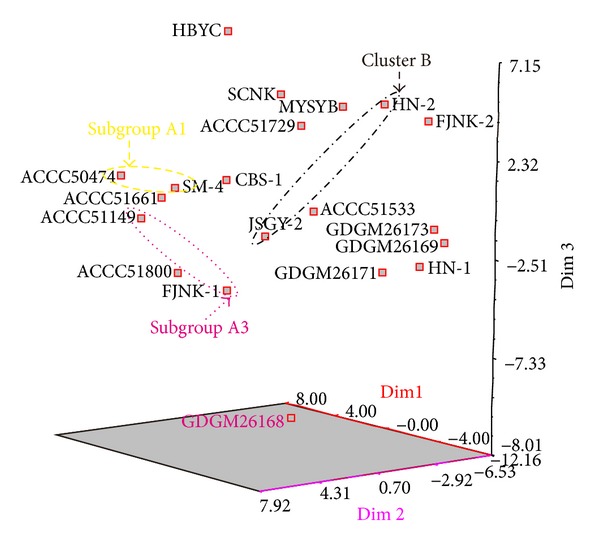
Three-dimensional map of principal coordinates analysis based TRAP data (black ellipse, cluster B; yellow ellipse, subgroup A1; pink ellipse, subgroup A3.e).

PCoA analysis based on the fragments of TRAP amplification was shown in Figure 3. The data indicated that the similar clusters were those with UPGMA. The three most informative PCoA components accounted for 35.48% of the variations observed (Figure 3). Most of the commercial cultivars (storage strains) could not cluster together to one group like the previous studies by ISSR and SRAP [13, 14].

The strains of* H. marmoreus* might be from two ancestors according to the result of phylogenetic analysis based on TRAP. The genetic diversity in cluster A was more polymorphic than that in cluster B; however, the strains in cluster A2 were different from the others (Figure 2). In the TRAP phylogenetic analysis, the clusters were not consistent with strain origin and the color of fruit body, as well as the previous studies with ISSR and SRAP [13, 14], possibly because TRAP technology focuses on polymorphism of the function genes, while ISSR and SRAP, respectively, focus on microsatellite diversities and intron and extron diversities. Compared with the previous studies [13, 14], most strains with white basidiocarps were clustered in a subbranch in this study, possibly because of the TRAP technique associating with the phenotypes controlled by some particular genes.

### 3.3. Sequence Analyses

Among the fragments amplified with TRAP, 60 fragments were recycled and cloned into pMD19-T vector. Thirty-six sequences were obtained and then submitted to GenBank. The accession numbers of TRAP sequences were from KC906193 to KC906227 and KC906229. The lengths of the sequences ranged from 308 to 1338 bp. Eighteen sequences could encode the unknown function proteins based on BLAST prediction and open reading frame (ORF) analysis by web-based SoftBerry (Supplementary File 2).

Sequences of KC906206, KC906216, KC906218, KC906224, and KC906225 differed from each other and were partial sequences of heat shock protein 70 (HSP70) predicted by three models of BLAST search (BLAST N, BLAST X, and tBLAST X), which suggested a high diversity in genes of HSP70 of* H. marmoreus*. Based on BLAST X search, some other sequences of TRAP could be predicted to encode HSP protein, glycoside hydrolase, and unknown proteins, which coincided with the fragments selected to design fixed primers. Although the fixed primers were designed from the genes of* H. marmoreus*, only a small part of TRAP sequences could correspond with the chosen gene segments. There might be two reasons for that: one is the arbitrary primers amplifying fragments from genomic section matching with sequences of primers and the other may be that the low annealing temperature in PCR amplified many unspecific fragments [42].

The results of this study showed that TRAP was more effective than the other molecular markers to evaluate the genetic diversities of* H. marmoreus *strains.

## 4. Conclusion

The TRAP analysis was successfully applied to study the genetic diversities among the* H. marmoreus* strains. A total of 36 gene sequences of* H. marmoreus* were obtained. The results of this study indicated that the strains of* H. marmoreus* have medium level of genetic diversity.

## Supplementary Material

Figure S1: Result of specific sequence by BLAST N
Figure S2: Result of specific sequence by BLAST X
Table S1: Information of the strains of *Hypsizigus marmoreus*
Table S2: Fixed and arbitrary primers used in the TRAP analysis of *Hypsizigus marmoreus*
Table S3: Information of the sequences used to designed specific primers for *Hypsizigus marmoreus*

## Figures and Tables

**Table 1:** Primer combinations and some genetic information generated by TRAP markers.

Primer combination	Total bands	Polymorphism bands	Percentage of polymorphism	Polymorphism information content	Marker index	Fragment range size
EF69R1-EM10	12	9	75.00	0.50	5.94	100–2000
EU94L1-EM9	12	5	41.67	0.50	5.96	150–1900
GQ76R1-EM1	8	4	50.00	0.46	3.70	300–1100
GQ76R1-EM2	8	3	37.50	0.38	3.05	170–1200
GQ76R1-EM4	6	4	66.67	0.50	3.00	150–2100
GQ76R1-EM5	11	7	63.64	0.49	5.38	100–1200
GQ76R1-EM6	9	6	66.67	0.50	4.46	100–750
GQ76R1-EM17	17	11	64.71	0.50	8.50	120–2100
GQ76R1-ME1	13	10	76.92	0.49	6.38	250–2000
GQ76R1-ME2	12	7	58.33	0.50	5.99	150–1700
GQ76R1-ME4	14	11	78.57	0.48	6.72	150–1900
GQ76R1-ME7	10	4	40.00	0.48	4.84	120–1800
GQ76R1-ME8	5	3	60.00	0.49	2.46	300–1200
GQ76R1-ME9	7	6	85.71	0.39	2.75	150–1200
GQ76R1-ME11	13	9	69.23	0.50	6.48	150–1800
GQ76R1-ME13	11	7	63.64	0.48	5.28	120–1700
JN00L1-EM5	16	13	81.25	0.50	7.99	100–1600
JN00L1-EM10	14	10	71.43	0.50	6.99	200–900
JN00L1-EM16	10	8	77.78	0.50	4.48	100–900
JN00L1-EM26	10	8	80.00	0.50	5.00	120–1000
JN00L1-ME6	4	2	75.00	0.50	2.00	150–500
JN00L1-ME11	6	2	33.33	0.32	1.92	150–500
JN00R1-EM2	13	11	84.62	0.49	6.41	120–1700
JN00R1-EM6	10	9	90.00	0.45	4.52	200–700
JN00R1-EM24	9	8	88.89	0.46	4.15	150–1000
JN00R1-ME2	11	9	81.82	0.50	5.45	100–1200
JN00R1-ME4	10	9	90.00	0.49	4.94	100–1100
JN00R1-ME6	7	7	100.00	0.50	3.49	300–1500
Total	**287**	**202**				
Minimum	4	2	33.33	0.32	1.92	
Maximum	17	13	100.00	0.50	8.50	
Mean	**10.27**	**7.21**	**69.73**	**0.48**	**4.94**	
