# Supplementary material for: Genetic Diversity Analysis of Hypsizygus marmoreus with Target Region Amplification Polymorphism
Source: ScientificWorldJournal. 2014 Jun 9;2014:619746. doi: 10.1155/2014/619746 (PMC4071973; doi:10.1155/2014/619746)
Supplement: Supplementary file 1 — Figure S1: Result of specific sequence by BLAST N Figure S2: Result of specific sequence by BLAST X Table S1: Information of the strains of Hypsizigus marmoreus Table S2: Fixed and arbitrary primers used in the TRAP analysis of Hypsizigus marmoreus Table S3: Information of the sequences used to designed specific primers for Hypsizigus marmoreus [file 619746.f1.pdf]

Figure S1 Result of specific sequence by BLAST N

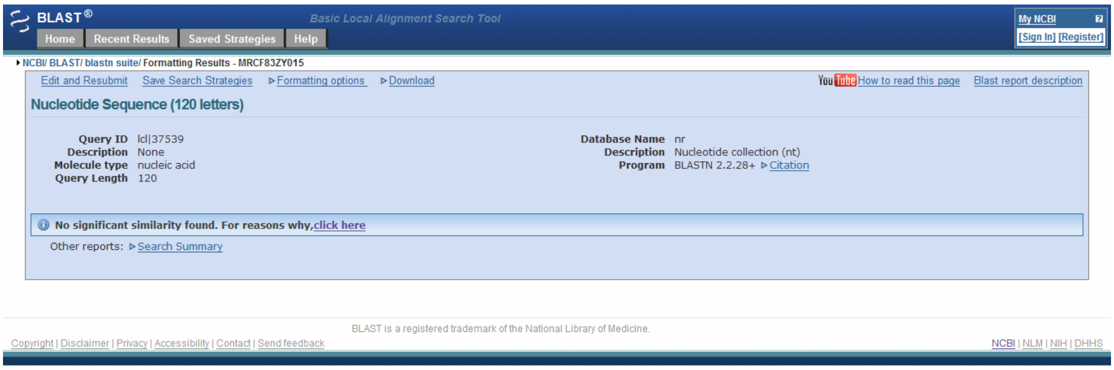

Figure S2 Result of specific sequence by BLAST X

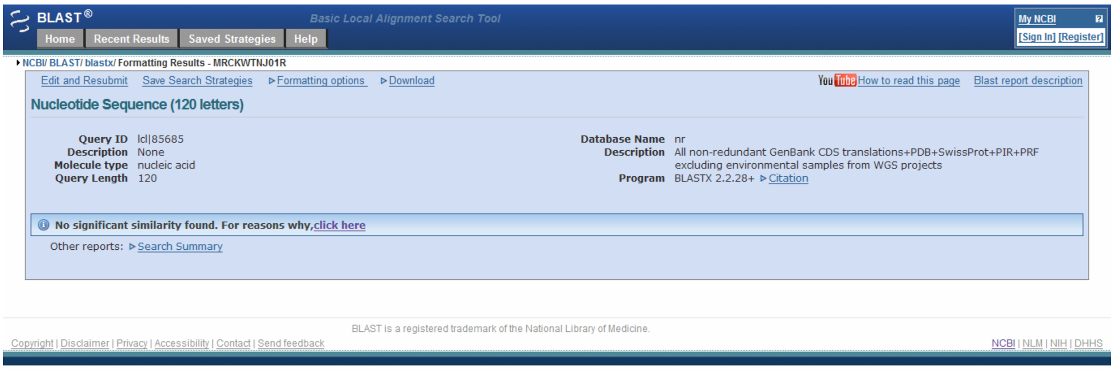

Table S1 Information of the strains of *Hypsizygus marmoreus*

No.	Strain Code	Origin	Strain Type
1	ACCC50474	Dalian University of Technology, Dalian, Liaoning, China	storage stain
2	ACCC51149	Xin Yu Edible Fungi Institute, Wuhan, Hubei, China	storage stain
3	ACCC51533	Institute of Agricultural Resources and Regional Planning, Chinese Academy of Agricultural Sciences, Beijing, China	storage stain
4	ACCC51661	Huan Yu Edible Fungi Institute, Jiayu, Hubei, China	storage stain
5	ACCC51729	Nanjing Agricultural University , Nanjing, Jiangsu, China	storage stain
6	ACCC51800	Shandong Agricultural University, Taian, Shandong, China	storage stain
7	GDGM26168	Shanghai Finc Bio-tech Inc., Shanghai, China	commercial cultivar
8	GDGM26169	Shandong Ronfun Group, Ltd., Dongying, Shandong, China	commercial cultivar
9	GDGM26171	Shanghai Tianchu Mushroom Co., Ltd., Shanghai, China	commercial cultivar
10	GDGM26713	Starway Bio-technology, Dongguan, Guangdong, China	commercial cultivar
11	FJNK-1	Fujian Academy of Agricultural Sciences, Fuzhou, China	commercial cultivar
12	FJNK-2	Fujian Academy of Agricultural Sciences, Fuzhou, China	commercial cultivar
13	GSGY-2	Gaoyou Institute of Edible Fungi, Gaoyou, Jiangsu, China	commercial cultivar
14	HN-1	Hunan Academy of Agricultural Science, Changsha, Hunan, China	commercial cultivar
No.	Strain Code	Origin	Strain Type
15	HN-2	Hunan Academy of Agricultural Science, Changsha, Hunan, China	commercial cultivar
16	HBYC	Yichang Kanghuiyuan Biology Technology Co., Ltd., Yichang, Hubei, China	commercial cultivar
17	SM-4	Sanming Mycological Institute, Sanming, Fujian, China	commercial cultivar
18	MYSYB	Mianyang Edible Fungi Research Institute of Sichuan, Mianyang, Sichuan, China	commercial cultivar
19	SCNK	Sichuan Academy of Agricultural Science, Chengdu, Sichuan, China	commercial cultivar

20	CBS-1	Changbaishan, Jilin, China	storage stain

Table S2 Fixed and arbitrary primers used in the TRAP analysis of *Hypsizygus marmoreus*

Primers	Sequence(5′→3′)	Origin
Fixed primers		
EF69R1	TTGTGAGCTTAGGCGTTA	*H. marmoreus* serine protease mRNA EF153269.2
GQ76L1	CGTTCCTGCATACTTCAA	*H. marmoreus* heat shock protein 70 GQ246176.1
GQ76R1	GCCTCTTCGATGAGATTC	*H. marmoreus* heat shock protein 70 GQ246176.1
EU94L1	CGGAAAGCGTTATAGGTT	*H. marmoreus* laccase mRNA EU375894.1
JN00R1	TCCTCGGTGTACTCAACA	*H. marmoreus*gene glyceraldehyde-3-phosphate dehydrogenase JN048800.1
JN00L1	GGGAGAAAGGGGTAGATT	
Arbitrary primers		
ME1	TGAGTCCAAACCGGATA	Li & Quiros (2001)
ME2	TGAGTCCAAACCGGAGC	
ME4	TGAGTCCAAACCGGACC	
ME6	TGAGTCCAAACCGGACA	
ME7	TGAGTCCAAACCGGACG	Budak et al.(2005)
ME8	TGAGTCCAAACCGGACT	
ME9	TGAGTCCAAACCGGAGG	
ME11	TGAGTCCAAACCGGAAC	
ME13	TGAGTCCAAACCGGAAG	
EM1	GACTGCGTACGAATTAAT	Li & Quiros (2001)
EM2	GACTGCGTACGAATTTGC	
EM4	GACTGCGTACGAATTTGA	

EM5	GACTGCGTACGAATTAAC	
EM6	GACTGCGTACGAATTGCA	
EM9	GACTGCGTACGAATTCAG	Budak et al.(2005)
EM10	GACTGCGTACGAATTCAT	
EM16	GACTGCGTACGAATTGTC	Ren et al (2012)
EM17	GACTGCGTACGAATTTAG	
EM24	GACTGCGTACGAATTATG	
EM26	GACTGCGTACGAATTCGG	

Table S3 Information of the sequences used to designed specific primers for *Hypsizigus marmoreus*.

Name	Sequence（3′-5′）	Length	Size of prediction
KC906199SP	Forward: CGA CGA ACG AGA CTG AAT	18	321
	Reverse: ATA CTG CGA AGA TGT GAT GA	20	
KC906204SP	Forward: TCA GAA CAT CCA GCG TAA G	19	323
	Reverse: ACC TCT ACC ATC CAC CAT T	19	
KC906207SP	Forward: TCA GAA CAT CCA GCG TAA G	19	323
	Reverse: ACC TCT ACC ATC CAC CAT T	19	
KC906214SP	Forward: ATG CTG AGT TGT TGA ATG C	19	190
	Reverse: GCC ATC CGA TTG CTA CAT	18	
KC906215SP	Forward: TTG CTG GTA TTT GGC TGT A	19	191
	Reverse: CGA CTG GAG TAA TGT GAA TAG	21	
KC906223SP	Forward: GCT TGT ATC CGT CGT GTA T	19	244
	Reverse: GGT AGT GGT AAC CTG AGT C	19	

GenbankAccess	sizeofsequen	composition of base	Blast N	Blast X	Putative conserved domains (size, function name	tBlast X	CDS	startandends	ORFs	startandends	AA number of

sion	ce							ize		ize	predicted protein
KC906193	909	267A202C217G223T	No significant similarity found	Laccaria bicolor S238N-H82 predicted protein partial mRNA, Accession: XP_001888969.1		Laccaria bicolor S238N-H82 hypothetical protein partial mRNA, Accession: XP_001888934.1	3	6-175/228-569/688-842	3	8-175/228-569/668-840	221

KC906194	564	193A1437C134G1000T	No significant similarity found	Trametes veriscolor FP-10664 SS1, accession: EIW51173.1/ Agaricus bisporus var. bisporus H97 predictal protein AGABI2DRAFT, accession: EKV42031.1		Agaricus bisporus clone FWBA165-D07, complete sequenc, accession: AC253814.1	1	46-537	1	46-537	164
KC906195	800	186A148C249G217T	No significant similarity found	Laccaria bicolor S238N-H82 predicted protein (Cover 22%)		Laccaria bicolor clone JGIAHZW-15X, complete sequence (Cover 21%)	1	61-300	1	61-300	80
KC906196	443	119A96C126G102T	Lepista nebularis isolate AFTQL-1D 1495 18S ribosomal RNA gene partial sequence/Richoniella asterospora 18 small subnit	Trametes versicolor FP-101664 SS1 checkpoint-link protein (CHK1), accession: EIW51416.1/ Medicago truncatula ATP synthase subunit beta, accession:		Richoniella asteropora 18S small subunit ribosomal RNA gene, partial sequence, accession JF76311.1/ unculturd Ascomycota clone BN4-51 18S ribosomal RNA gene,	0				

			ribosomal RNA gene, partial sequence	XP_003627732.1		partial sequence, accession GQ404768.1					
KC906197	708	180A179C154G195T	Serpula lacrymans var. lacrmans S7.3 hypothetical protein (Cover 22%)	No significant similarity found		Zebrafish phospholipase mRNA (Cover 30%)	0				
KC906198	308	95A72C63G78T	No significant similarity found	Laccaria bicolor S238N-H82 predicted protein partial mRNA, Accession: XP_001889811.1 (XP_001889801.1)		Laccaria bicolor S238N-H82 predicted protein partial mRNA, Accession: XP_001889811.1 (XP_001889801.1)	1	146-237	1	146-237	30
KC90619	909	129A112C11	No significant similarity found	No significant similarity found		No significant similarity found	0				

9		6G1112T									
KC90620 0	469	129A1112C1116G1112T	No significant similarity found	Moniliophihora permiciosa FA553 hypothetical protein MPER_13238, accession: XP_002387823.1	RF+3: 42-200, EYA-cons_domain	Pinus taeda clone PT_7Ba2882K18, complete sequence, accession: AC241291.1	0				
KC906201	1075	275A272C270G258T	Hypsizygus marmoreus heat shock protein 70 (HSP70) mRNA complete cds, Accession: GQ246176.1	Hypsizygus marmoreus heat shock protein 70, Accession: ACT33635.1	RF+2: 10-1035, NAD-GH (superfamilies); RF-3: 575-1060, NBD_sugar-kinase_HSP70_action superfamily;1-1065, dnaK (Multi-domain)	Hypsizygus marmoreus heat shock protein 70 (HSP70) mRNA complete CDS, Accession: GQ246176.1	1	62-1075			303
KC9062	1338	305A386C	Laccaria bicolor S238N-H82 predicted protein (Query cover	No significant similarity found	RF-3: 500-600, Bactofilin	Laccaria bicolor S238N-H82 predicted protein partial mRNA (Query cover	1	140-97			194

02		316G331T	39%)			36%)		0			
KC906203	510	124A126C126G134T	No significant similarity found	Agaricus bisporus var. burnethii hypothetical protein AGABI2DEAFT_182914, accession: EKM82322.1		Laccaria bicolor S238N-H82 hypothetical protein partial mRNA, accession: XM_001994024.1	0				
KC906204	927	267A212C245G203T	No significant similarity found	No significant similarity found		No significant similarity found	0				
KC90620	893	243A197C21	No significant similarity found	Laccaria bicolor S238N-H82 glycoside hydrolase family 47 protein, accession:	RF-1: 740-550, Glyco_hydro_47 Superfamily	No high similarity sequence over 25%	1	65-789			152

5		9G234T		XP_001881296.1							
KC906206	1074	274A272C272G256T	Hypsizygus marmoreus heat shock protein 70 (HSP70) mRNA complete cds, Accession: GQ246176.1	Hypsizygus marmoreus heat shock protein 70, Accession: ACT33635.1	RF+3: 10-1065, NAD-GH (superfamilies); RF-2: 560-1075, NBD_sugar-kinase_HSP70_actin superfamily; RF-2: 1-1070, dnaK (Multi-domains)	Hypsizygus marmoreus heat shock protein 70 (HSP70) mRNA complete CDS, Accession: GQ246176.1	1	63-1704			302
KC906207	955	270A221C253G211T	No significant similarity found	No significant similarity found		No significant similarity found	0				
KC906	757	172A191	No significant similarity found	No high similarity sequence over 29%		No significant similarity found	0				

208		C170G224T									
KC906209	807	191A203C214G199T	No significant similarity found	Lacaria bicolor S238N-H82 predicated protein, accession: XP_001880772.1	RF+3: 40-425, Anticodon_Ia_like superfamily (anticodon binding site & tRNA binding surface)	Lacaria bicolor S238N-H82 hyprothetical protein partial mRNA, accession: XM_001880737.1	1	41-797			252
KC906210	758	192A210C146G210T	Clitocybe candicans isolate AFTOL-1D 541 18 ribosomal RNA gene, partial sequence, Accession: AY771609.1	Acinetobacter sp. P8-3-8 hypothetical protein, accession: ZP_0914283.1		Psilocybe montana isolate AFTOL-1D 820 18S ribosomal RNA gene, partial sequence, accession: DQ465342.1	0				
KC90662	669	202A160C	No significant similarity found	Trametes versicolor FP-101664 SS1 hypothetical protein TRAVEDRAF		No high similarity sequence over 40%	0				

11		193G114T		T_146600, accession: EIW60909.1							
KC9062112	1061	337A179C200G345T	No significant similarity found	Laccaria bicolor S238N-H82 predicted protein, accession: XP_001883620.1		No high similarity sequence over 40%	0				
KC9062113	779	221A180C201G177T	No significant similarity found	Laccaria bicolor S238N-H82 glycoside hydrolase family 5 protein, accession: XP_001879429.1/ Volvarella volvacea endoglucanase, accession: ACE06751.1		Volariella volvacea endoglucanase (egl) mRNA complete cds, accession: EU716328.1	1	184-490			82
KC9062	868	203A194C	No significant similarity found	No significant similarity found		No significant similarity found	0				

14		251G220T									
KC906215	336	82A84C87G83T	No significant similarity found	No significant similarity found		No high similarity sequence covered 50%	0				
KC906216	1074	274A272C272G256T	Hypsizygus marmoreus heat shock protein 70 (HSP70) mRNA complete cds, Accession: GQ246176.1	Hypsizygus marmoreus heat shock protein 70, Accession: ACT33635.1	RF+3: 10-1045, NAD-GH (superfamilies); RF-2: 562-1065, NBD_sugar-kinase_HSP70_actin superfamily; RF-2: dnaK (Multi-domains)	Hypsizygus marmoreus heat shock protein 70 (HSP70) mRNA complete CDS, Accession: GQ246176.1	1	63-1704			302
KC906217	302	79A71C89G63T	No significant similarity found	Laccaria bicolor S238N-H82 predicted protein, accession: XP_001879074.1	RF-2: 183-273, mrel1; RF-3: 53-123, mrel1	Laccaria bicolor S238N-H82 predicted protein, accession: XP_001879039.1	0				

KC906218	1074	275A272C271G256T	Hypsizygus marmoreus heat shock protein 70 (HSP70) mRNA complete cds, Accession: GQ246176.1	Hypsizygus marmoreus heat shock protein 70, Accession: ACT33635.1	RF+3: 10-1045, NAD-GH (Superfamilies); RF-2: 555-1071, NBD_sugar-kinase_HSP70_actin superfamily; RF-2: 1-1074, dnaK (Multi-domains)	Hypsizygus marmoreus heat shock protein 70 (HSP70) mRNA complete CDS, Accession: GQ246176.1	1	63-1704			302
KC906219	745	170A203C178G194T	No significant similarity found	Coprinopsis cinere F-box/WD repeat-containing protein 11, accession: XP_001832905.2 / Stereum hirsutmu FP-91666 SS1 WD40 repeat-like protein, accession EIM89687.1	RF+1: 235-700, WD40 superfamily (structural tetrad); RF+1: 235-700, COG2319 (Multi-domains)	No significant similarity found	0				
KC906222	1146	269A286C28	No significant similarity found	Sterpula lacrymans var. lacrymans S7.9 hypothetical protein SERLADRAFT_443947,	RF-2: 75-445, OTU-domain-containing protein (Superfamilies)	No significant similarity found	1	48-980			270

0		9G302T		accession: EGO18612.1						
KC906221	425	113A1107C113G97T	No significant similarity found	Laccaria bicolor S238N-H82 predicted protein, accession: XP_001874648.1 / Agaricus bisporus var. burnettii JB 137-S8 hypothetical protein AGABI2DRAFT_54902, accession EKM81845.1	RF-1: 320-423, snRNP component HSH155 (Multi-domains); RF-2: 5-272, snRNP component HSH155 (Multi-domains)	Agaricus bisporus clone FWBA189-L19, complete sequence, accession: AC253834.1	0			
KC906222	1352	359A319C309G365T	No significant similarity found	Agaricus bisporus var. bisporus H97 hypothetical protein AGABI2DRAFT_141320, accession: EKV50458.1	RF=1: 10-250, MutS_I (Superfamilies)	No significant similarity found	1	49-1315		343
KC90	520	149A11	No significant similarity	No significant similarity found		No significant similarity found	1	63-1		44

6223		5C1266G1300T	found					99			
KC9006224	1074	274A274C271G255T	Hypsizygus marmoreus heat shock protein 70 (HSP70) mRNA complete cds (GQ246176.1)	Hypsizygus marmoreus heat shock protein 70, Accession: ACT33635.1	RF+3: 10-1050, NAD-GH; RF-2: 565-1060, NBD_sugar-kinase_HSP70_actin superfamily; 460-535, dnaK (Multi-domains)	Hypsizygus marmoreus heat shock protein 70 (HSP70) mRNA complete CDS, Accession: GQ246176.1	1	63-1074			302
KC9006225	1074	255A272C274G273T	Hypsizygus marmoreus heat shock protein 70 (HSP70) mRNA complete cds, Accession: GQ246176.1	Hypsizygus marmoreus heat shock protein 70, Accession: ACT33635.1	RF+2: 1-505, NBD_sugar-kinase_HSP70_actin superfamily; 545-605, dnak (Mult-domains); RF-3: 25-1060, NAD-GH	Hypsizygus marmoreus heat shock protein 70 (HSP70) mRNA complete CDS, Accession: GQ246176.1	1	1-1012			302
KC90	647	189A18	Laccaria bicolor S238N-H82 hypothetical	Laccaria bicolor S238N-H82 predicted protein,	RF-3: 305-625, thymidylate synthase_P	No high similarity sequence over	0				

6226		0C147G131T	protein partial mRNA, Accession: XM_001889459.1	accession: XP_001889494.1	yrimidine_HMase superfamily (Mult-domain)	50%					
KC906227	647	132A147C180G188T	Laccaria bicolor S238N-H82 hypothetical protein partial mRNA, Accession: XM_001889459.1	Laccaria bicolor S238N-H82 predicted protein, accession: XP_001889494.1	RF+2: 15-345, thymidylate synthase_Pyrimidine_HMase superfamily (Multi-domain)	No high similarity sequence over 53%	0				
KC906229	440	114A100C99G127T	No significant similarity found	Agaricus bisporus var burnettii JB137-S8 hypothetical protein, accession: EKW80790.1 / Agaricus bisporus var. bisporus H97, accession: EKV47257.1	RF+2: 125-425, CypX superfamily	Coprinosis cinerea okayama 7#130 SeqLit hypothetical protein, mRNA, accession: XM_001830362.2	1	155-429			78
